# Skin tests may induce DRESS relapse

**DOI:** 10.1186/2045-7022-4-S3-P136

**Published:** 2014-07-18

**Authors:** Benoit Ben Said, Frederic Berard, Florence Hacard, Pauline Pralong, Brigitte Balme, Jean Francois Nicolas

**Affiliations:** 1 Chu Lyon Sud Clinical Immunology and Allergology Department, Inserm U111, Universite Lyon 1, France; 2 Chu Lyon Sud , Inserm U111, Universite Lyon 1, Clinical Immunology and Allergology Department, France; 3 Chu Lyon Sud, Clinical Immunology and Allergology Department, France; 4 Chu Grenoble, Dermatology Department, France; 5 Chu Lyon Sud, Pathology Department, France

## 

DRESS is a rare but life threatening drug allergic disease. The value of skin tests, especially patch-tests (PT) for defining the culprit drug were recently reported. Nevertheless DRESS has been associated with high risk of disease flare linked to drug rechallenge (culprit drug or not) or viral reactivation . In this study we analyzed the frequency of patch test-induced DRESS flares. Method Between 01/2009 and August 2013, patients with DRESS syndrome (diagnosis score KARDAUN >5 : definite case) were hospitalized for drug allergic evaluation and received skin patch tests (Patch tests and/or IDT) with 72h reading. In case of DRESS flare blood viral load (HHV6, EBV, CMV), white blood cells count , liver enzymes and kidney function were performed. Results 68 DRESS were included. Among them, 39 patients showed positive PT (57%) and 29 were negative at the 72h reading. From the 39 PT positive patients, 8 patients (20%) experienced a DRESS flare in the form of a mild skin rash developing in the 48h following the realization of skin tests. The rash started before the 72h reading in all cases. In two cases, general signs were noted and in one case eosinophilia was found (760/mm3 N<500) but no patients reached the KARDAUN score >5. Liver and kidney assays were normal. Viral blood PCR for herpes viruses were negative at the onset of the relapse and during the two following days. The culprit drugs were dominated by betalactam antibiotics (4/8 : 50%). Skin biopsies of the skin rash were compatible with a delayed hypersensitivity reaction in all cases. Interestingly 4 patients (50%) were able to induce non specific rash after introducing drugs unrelated to the culprit one with negative blood herpes viral load. Conclusion Our study demonstrates that positive skin tests may induce a mild reactivation of DRESS in a noticeable proportion of patients. The delay between skin tests and flare (in all cases <48h) confirms that DRESS is a drug specific delayed hypersensitivity reaction. The negativity of viral load does not confirm the relation of DRESS flare with viral reactivation. Many hypotheses could explain the occurrence of skin test-induced DRESS rash in these patients : T reg dysfunction after DRESS syndrome , very strong T cell sensitization in these patients, long term persistence of drug in skin, T cell chronic pre-activation state, as suggested by the frequency of non specific DRESS flare following alternative drug administration. Clinicians should be aware of the risk of skin test-induced DRESS flares in order to optimize the management of these patients during the allergological work up.

